# Effect of Melatonin on Human Dental Papilla Cells

**DOI:** 10.3390/ijms151017304

**Published:** 2014-09-26

**Authors:** Ryusuke Tachibana, Seiko Tatehara, Shuku Kumasaka, Reiko Tokuyama, Kazuhito Satomura

**Affiliations:** Department of Oral Medicine and Stomatology, School of Dental Medicine, Tsurumi University, 2-1-3 Tsurumi, Tsurumi-ku, Yokohama, Kanagawa 230-8501, Japan; E-Mails: tachibana-r@tsurumi-u.ac.jp (R.T.); tatehara-s@tsurumi-u.ac.jp (S.T.); kumasaka-shuku@tsurumi-u.ac.jp (S.K.); tokuyama-r@tsurumi-u.ac.jp (R.T.)

**Keywords:** melatonin, melatonin 1a receptor, DP-805 cells, dental papilla cell, tooth development, human

## Abstract

Melatonin regulates a variety of biological processes, which are the control of circadian rhythms, regulation of seasonal reproductive function and body temperature, free radical scavenging and so on. Our previous studies have shown that various cells exist in human and mouse tooth germs that express the melatonin 1a receptor (Mel1aR). However, little is known about the effects of melatonin on tooth development and growth. The present study was performed to examine the possibility that melatonin might exert its influence on tooth development. DP-805 cells, a human dental papilla cell line, were shown to express Mel1aR. Expression levels of mRNA for Mel1aR in DP-805 cells increased until 3 days after reaching confluence and decreased thereafter. Real-time reverse transcription-polymerase chain reaction showed that melatonin increased the expression of mRNAs for osteopontin (OPN), osteocalcin (OCN), bone sialoprotein (BSP), dentin matrix protein-1 (DMP-1) and dentin sialophosphoprotin (DSPP). Melatonin also enhanced the mineralized matrix formation in DP-805 cell cultures in a dose-dependent manner. These results strongly suggest that melatonin may play a physiological role in tooth development/growth by regulating the cellular function of odontogenic cells in tooth germs.

## 1. Introduction

Melatonin, a hormone synthesized and secreted from the pineal gland mainly at night, was isolated and purified from the bovine pineal gland by Lerner *et al* [1]. Its secretion is controlled by the light:dark cycle in such a manner that it is released in large amounts during the night, but only minimally during the day [2,3]. Recently, this hormone was shown to be synthesized by other tissues, such as retina [4], lens [5], ovarium [6], intestine [7] and salivary glands [8]. Melatonin plays an important role in many physiological processes including control of circadian rhythms [9], regulation of body temperature [10], blood pressure [11], and seasonal reproductive function [12,13,14], free radical scavenging [15,16], and oncostatic activity [17,18,19].

Recently, the biological actions of melatonin in the oral cavity have received attention. Melatonin has been investigated in relation to periodontal disease, bone remodeling, osseointegration of dental implant, and oral cancer [20,21,22,23]. In particular, it has become clear that melatonin regulates bone remodeling. Melatonin effects on bone formation include stimulation of the proliferation and differentiation of osteoblasts and promotion of the mineralization of the extracellular matrix *in vitro* [24,25,26]. In addition, the administration of melatonin in mice increases the volume of newly-formed cortical bone of the femora [27]. Moreover, in respect to the effect on bone resorption, melatonin suppresses osteoclast differentiation by reducing mRNA expression of receptor activator of NF-κB (RANK) and by increasing the expression of osteoprotegerin [28,29]. Furthermore, melatonin adjusts the bone metabolism indirectly through the systemic hormone such as parathyroid hormone (PTH), calcitonin and estrogen [30]. Recent studies suggest that melatonin may be related to tooth development as well as bone tissue [31,32].

We reported for the first time that ameloblasts, stratum intermedium cells, stellate reticulum cells, and odontogenic epithelial cells in human tooth germs of mandibular third molars expressed the melatonin 1a receptor (Mel1aR), which is the most ubiquitous of the membrane receptors of melatonin. The expression level of mRNA for Mel1aR in HAT-7 cells, a rat odontogenic epithelial cell line, increased after reaching confluence *in vitro*. These results suggest that melatonin has some effect on tooth formation/growth [31]. Another study indicated that melatonin suppressed the proliferation of rat dental papilla cells in a dose-dependent manner and promotes the differentiation [32]. However, the effects of melatonin on human odontogenic cells still remain poorly defined.

Thus, to examine whether melatonin is involved in human tooth development, in the present study we investigated the effects of melatonin on proliferation and differentiation of a human dental papilla cell line, DP-805.

## 2. Results

### 2.1. Localization of Melatonin 1a Receptor (Mel1aR) in Human Tooth Germs

**Figure 1:**
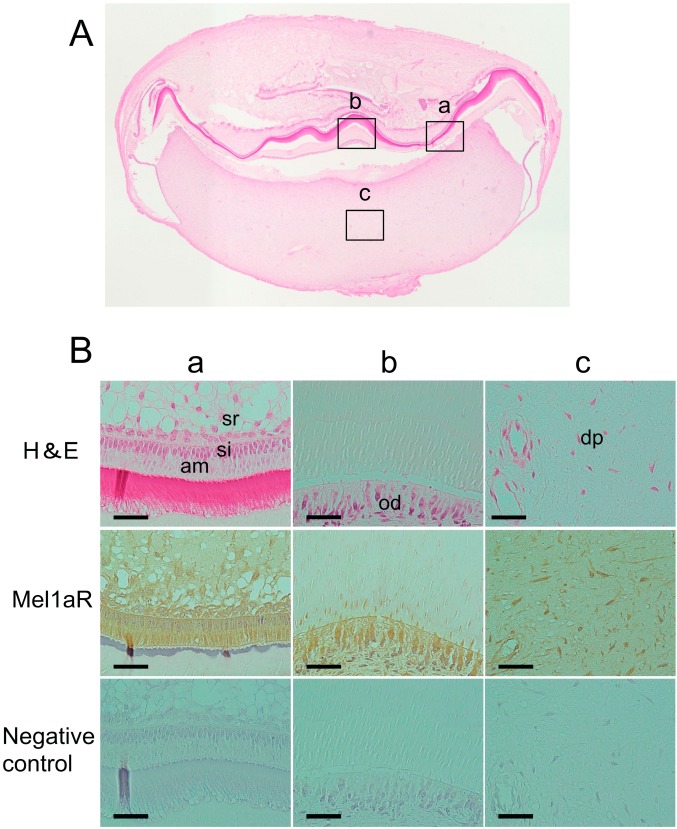
(**A**) Transverse section of a human tooth germ stained with hematoxylin and eosin (H&E). Higher magnification views of several portions of the tooth germ indicated by alphabetized squares were shown in panel B; and (**B**) Higher magnification views of characteristic areas of the tooth germ. Mel1aR was noted to be expressed in ameloblasts (am), stratum intermedium cells (si), stellate reticulum cells (sr), odontoblasts (od) and dental papilla cells (dp). H&E: hematoxylin and eosin staining. Mel1aR: 3,3'-diaminobenzidine (DAB) and hematoxylin staining. Negative control: normal mouse IgG. Scale bars = 25 µm.

General histology of the tooth germs of human mandibular third molars at the late bell stage showed an initial formation of dentin and enamel in the area where the cusp of the crown would later be formed (Figure 1A). To examine whether Mel1aR is expressed in human and rat tooth germs, immunohistochemical analyses for Mel1aR were performed. These analyses revealed that the ectomesenchymal cells in the human tooth germs, *i.e.*, the odontoblasts and the dental papilla cells, were positive for Mel1aR (Figure 1B(a–c)).

### 2.2. Localization of Mel1aR in Rat Tooth Germs

**Figure 2:**
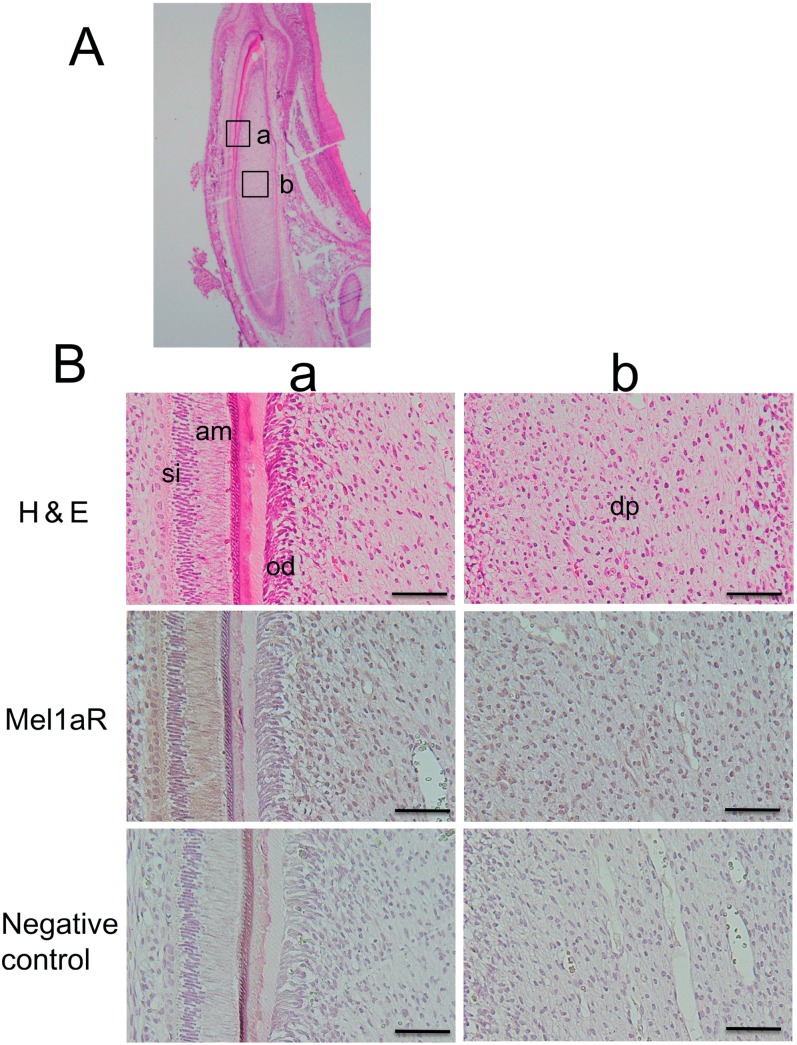
(**A**) Transverse section of a rat tooth germ stained with hematoxylin and eosin (H&E). Higher magnification views of several portions of the tooth germ indicated by alphabetized squares were shown in panel B; and (**B**) High magnification views of characteristic areas of the tooth germ. Mel1aR was expressed in ameloblasts (am), stratum intermedium cells (si), odontoblasts (od), dental papilla cells (dp). H&E: hematoxylin and eosin staining. Mel1aR: DAB and hematoxylin staining. Negative control: normal muse IgG. Scale bars = 25 µm.

Next, we confirmed the immunolocalization of Mel1aR in the rat tooth germs of incisors. Similar to the human tooth germs, ameloblasts, stratum intermedium cells, stellate reticulum cells, odontoblasts, and dental papilla cells were noted to be positive for Mel1aR (Figure 2). This staining pattern was substantially similar to that in human tooth germs.

### 2.3. Expression of Mel1aR in Human Dental Papilla Cells

**Figure 3:**
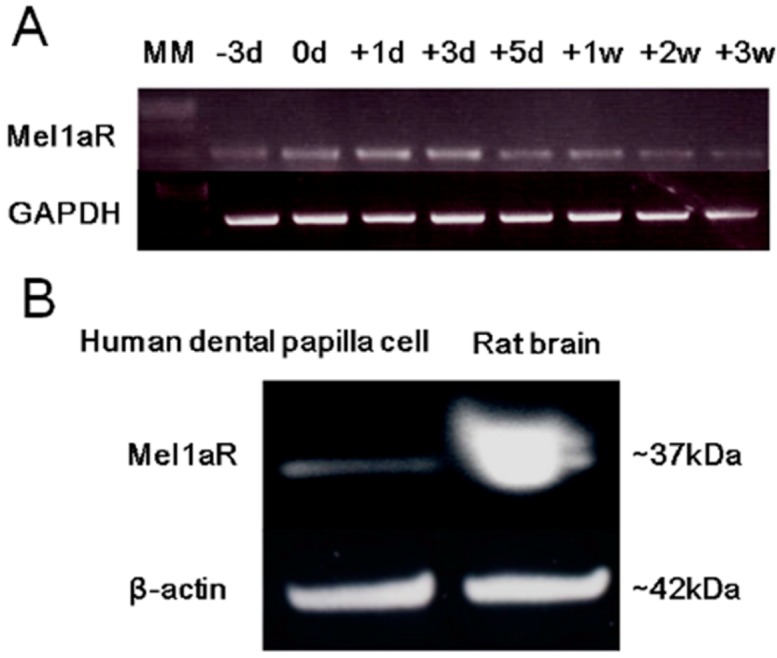
(**A**) The expression of mRNA for Mel1aR in DP-805 cells (a human dental papilla cell line). Mel1aR mRNA expression was detected at 3 days of culture before reaching confluence (−3d), gradually increased until 3 days after reaching confluence (+3d), and gradually decreased thereafter until 3 weeks of culture (+3W); and (**B**) Expression of Mel1aR protein in DP-805 cells. DP-805 cells were cultured until reaching confluence. Thereafter, the expression of Mel1aR protein was analyzed by Western blot. A major band for Mel1aR was observed at ~37 kDa. The amount of protein loaded for DP-805 cells and rat brain was 80 and 100 µg, respectively.

The expression of mRNA for Mel1aR in a human dental papilla cells line, DP-805, was confirmed using semi-quantitative RT-PCR. The expression level of Mel1aR mRNA increased until 3 days after reaching confluence and then gradually decreased with time (Figure 3A). Next, to examine the expression of Mel1aR protein in DP-805 cells, Western blot analysis for Mel1aR was analyzed. This analysis also confirmed the expression of Mel1aR protein in DP-805 cells (Figure 3B).

### 2.4. Effect of Melatonin on the Proliferation of Human Dental Papilla Cells

**Figure 4:**
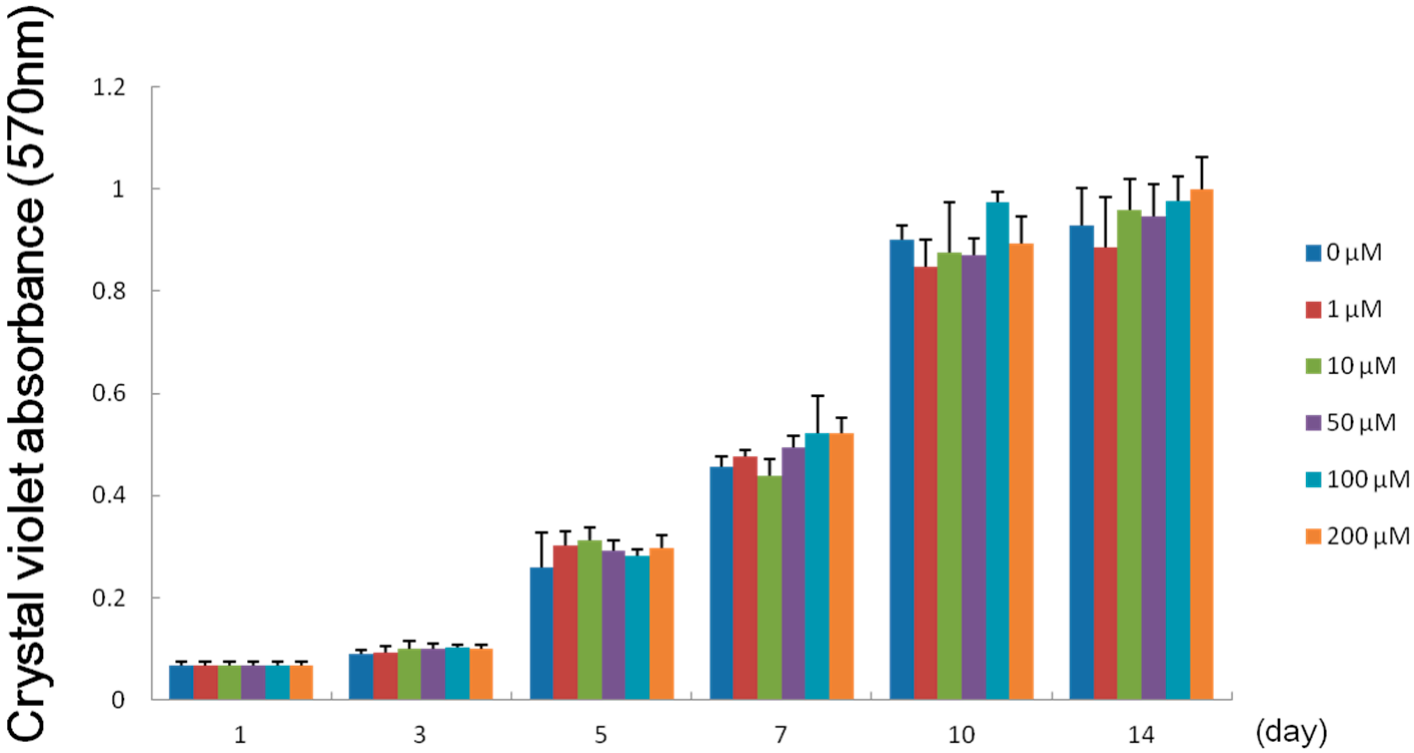
Effect of melatonin on the proliferation of DP-805 cells. DP-805 cells were seeded into 24-well culture plates at a cell density of 1 × 10^4^ cell/well and cultured in α-modified Eagle’s Medium (α-MEM) containing 1% fetal bovine serum (FBS) and a variety of concentrations (0, 1, 10, 50, 100 and 200 µM) of melatonin. The proliferation of DP-805 cells was analyzed using a crystal violet staining method. Melatonin exerted no influence on the proliferation of DP-805 cells at any concentrations not less than 1 µM and not more than 200 µM. Values are the mean ± SD.

To investigate the effect of melatonin on the proliferation of human dental papilla cells, DP-805 cells were cultured in the absence or the presence of various concentrations of melatonin (1, 10, 50, 100 and 200 µM). Melatonin exerted no significant influence on the proliferation of DP-805 cells at concentrations examined (Figure 4).

### 2.5. Effect of Melatonin on the Differentiation of Human Dental Papilla Cells

**Figure 5:**
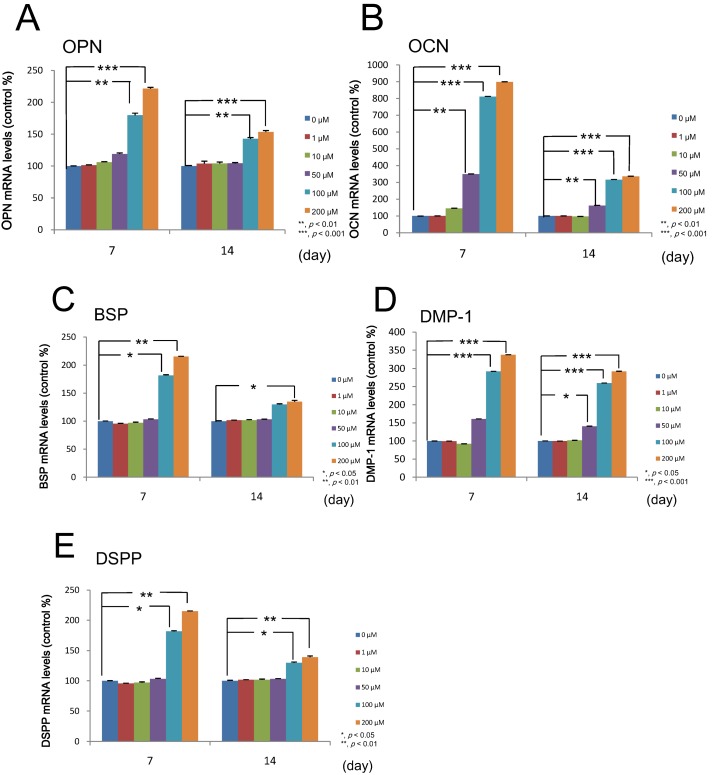
Effect of melatonin on the differentiation of DP-805 cells. DP-805 cells were plated at a cell density of 1 × 10^5^ cells/well and cultured until they reached confluence. Thereafter, the cells were treated with various concentrations (0, 1, 10, 50, 100 and 200 µM) of melatonin for 14 days. Melatonin promoted the expression of mRNA for osteopontin (OPN) (**A**); bone sialoprotein (BSP) (**C**); dentin matrix protein-1 (DMP-1) (**D**) and dentin sialophosphoprotin (DSPP) (**E**) at concentrations equal to and more than 100 µM; The expression of mRNA for osteocalcin (OCN) (**B**) was also enhanced at concentrations of at least 50 µM. Values are expressed as the mean ± SEM. * *p* < 0.05, ** *p* < 0.01, *** *p* < 0.001.

Using quantitative real-time RT-PCR, we examined the effects of melatonin on the expressions of mRNA for odontogenic makers, *i.e.*, osteopontin (OPN), osteocalcin (OCN), bone sialoprotein (BSP), dentin matrix protein-1 (DMP-1) and dentin sialophosphoprotin (DSPP) in DP-805 cells treated with various concentrations (0, 1, 10, 50, 100 and 200 µM) of melatonin. As a result, melatonin was proven to enhance the expressions of mRNA for these odontogenic maker genes (Figure 5).

### 2.6. Effect of Melatonin on the Mineralized Matrix Formation by Human Dental Papilla Cells

**Figure 6:**
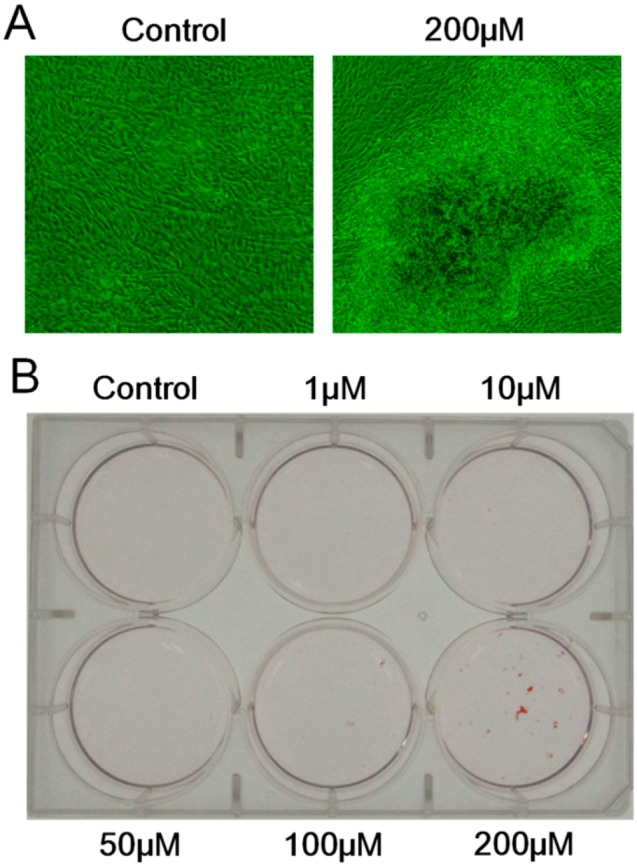
The effect of melatonin on mineralized matrix formation by DP-805 cells. DP-805 cells were seeded in 6-well culture plates at a cell density of 1 × 10^5^ cells/well. The cells were treated with various concentrations (0, 1, 10, 50, 100 and 200 µM) of melatonin for 28 days. The phase-contrast images showed that DP-805 cells cultured in the presence of 200 µM melatonin formed mineralized matrix (**A**); and The extracellular matrix mineralization was evaluated by Alizarin Red S staining (**B**).

To elucidate whether melatonin exerts its influence on the mineralized matrix formation by human dental papilla cells, the cultures of DP-805 cells with or without various concentrations (0, 1, 10, 50, 100 and 200 µM) of melatonin were analyzed by Alizarin Red S staining method. Mineralized matrix formation was observed only in the cultures treated with 200 µM of melatonin (Figure 6).

## 3. Discussion

In the present study, we attempted to elucidate the possible biological role of melatonin in tooth development. As the first step for this purpose, immunohistochemical analysis was performed to examine whether Mel1aR, the most potent membrane receptor for melatonin, was expressed in human and rat tooth germs. As a result, Mel1aR was expressed in a variety of odontogenic cells in the tooth germs at the late bell stage of tooth development. Importantly, the immunolocalization pattern of Mel1aR in the tooth germs was common between human and rat. This expression pattern was also consistent with findings reported in the previous study by Kumasaka *et al.* [31]. This similarity in the expression pattern of melatonin strongly suggest the possibility that melatonin does exert its influence on the tooth development.

Based on these results, we examined melatonin’s effect on dental papilla cells, a major cell population of ectomesenchymal origin which differentiate into dentin-forming odontoblasts, by using a human dental papilla cell line, DP-805 cells. RT-PCR analysis and Western blot analysis showed that DP-805 cells expressed the mRNA and protein of Mel1aR, respectively. Interestingly, the expression levels of mRNA for Mel1aR gradually increased until 3 days after reaching confluence and then gradually decreased. This finding was considered to suggest that melatonin might exert its influence on early differentiation rather than the proliferation of dental papilla cells. Consistent with this speculation, melatonin was proven to have no significant effect on the proliferation of DP-805. In contrast, melatonin promoted the expression of mRNA for odontoblastic differentiation markers such as OPN, OCN, BSP, DMP-1 and DSPP at concentrations of 50 to 200 µM. Additionally, melatonin enhanced mineralized matrix formation by DP-805 cells. These results clearly revealed that melatonin exerted positive effects not on the proliferation, but the odontoblastic differentiation of human dental papilla cells. The previous report by Liu *et al.* [32] also showed that melatonin promoted the differentiation of rat dental papilla cells. Our findings are consistent with the results reported in this study. As to melatonin’s effect on cell proliferation, it was reported that melatonin reduced the cell growth of rat dental papilla cells in a dose-dependent manner [28]. In contrast to this, our investigation detected no significant effects on the proliferation of human dental papilla cells. Although the reason for this discrepancy remains unclear in the present study, it appears that melatonin has no positive effect on the proliferation of dental papilla cells.

Melatonin actions are mediated by its binding to specific membrane receptors including Mel1aR. However, the above-mentioned report demonstrated that melatonin affects the rat dental papilla cells by binding mitochondrial membranes, without the pathway of the melatonin receptor [32]. Based on these facts, the mechanism by which melatonin promotes the differentiation of human dental papilla cells should be investigated carefully in future studies to resolve this issue. On the other hand, quite a few studies reported that the effects of melatonin on cell proliferation and differentiation depend on the cell types and melatonin concentrations [25,26,27,33]. Consequently, for this reason, the biological actions of melatonin should be multidirectionally investigated with caution in future studies.

In general, melatonin concentration in peripheral blood reaches the highest levels between the ages of 4 and 7 years [34], and thereafter gradually declines until about 12 years old [35]. Importantly and interestingly, the permanent teeth crowns are formed at the age of 4 to 8 years. In this stage of tooth development, enamel and dentin are actively being formed by ameloblasts and odontoblasts, respectively. These facts can also easily cause us to imagine that melatonin might indeed exert its influence on enamel/dentin formation by ameloblasts/odontoblasts *in vivo* as well as *in vitro*. In fact, in the previous study, we showed that Mel1aR was expressed by both ameloblasts and odontoblasts. Histomorphometric analysis of tooth formation under the administration of exogenous melatonin would be very helpful to elucidate and to confirm the physiological role of melatonin in tooth development.

## 4. Experimental Section

### 4.1. Human Tissue Samples

The tooth germs of the mandibular third molars in the late bell stage, which were extracted from five Japanese boys and girls (9 or 10 years of age) during orthodontic treatment, were used in this study. The experimental protocol was approved by the Ethical Review Committee of the Tokushima University Hospital (approval number: 542, approval date: 23 June 2008) and informed consent was obtained for the use of the tooth germs. Extirpated tooth germs were fixed with 10 N Mildform^®^ (Wako Pure Chemical Industries, Ltd., Osaka, Japan), decalcified in 10% EDTA (Wako), and embedded in paraffin. Sections 5 µm thick were cut, deparaffinized and stained with hematoxylin and eosin. The data presented herein are representative of reproducible results from five different donors.

### 4.2. Rat Tissue Samples

The housing care and experimental protocol were approved by the Animal Care and Use Committee of the School of Dental Medicine, Tsurumi University (approval number: 24A052, approval date: 25 January 2013). The mandibles of 3-day-old F344 rats (Clea Japan, Tokyo, Japan) were extirpated, fixed with 10 N Mildform^®^ (Wako), decalcified briefly in 10% EDTA (Wako) and embedded in paraffin. Sections 3 µm thick were cut, deparaffinized and stained with hematoxylin and eosin.

### 4.3. Immunohistochemistry

Sections of human tooth germs and rat mandibles were transferred onto poly-l-lysine-coated glass slides. After deparaffinization with xylene and rehydration with descending concentrations of ethanol, endogenous peroxidase was blocked by treatment with 3% H_2_O_2_ in methanol for 1 h at room temperature (RT). After treatment with 20% normal goat serum (Nichirei Corporation, Tokyo, Japan) at RT for 1 h, sections were incubated with the primary antibody (mouse anti-melatonin 1a receptor monoclonal antibody; Abnova Corporation, Taipei, Taiwan) diluted 1:1000 in phosphate-buffered saline (PBS, pH 7.4) containing 1% bovine serum albumin at 4 °C overnight. After washing with PBS, the localization of Mel1aR was visualized using a Histofine SAB-PO(M) Kit (Nichirei) and a 3,3'-diaminobenzidine (DAB) Substrate Kit (Nichirei). Sections were counterstained with hematoxylin and mounted. The specificity of the immunoreaction was confirmed by incubation with normal mouse IgG (Nichirei) instead of the primary antibody.

### 4.4. Cell Culture

DP-805 cells, a human dental papilla cell line originating from tooth germ of mandibular third molar at the late bell stage of a 14-year-old Japanese girl who underwent a germectomy for orthodontic treatment, was obtained from the First Department of Oral and Maxillofacial Surgery School of Dentistry, The University of Tokushima (Tokushima, Japan). DP-805 cells were cultured in α-modified Eagle’s Medium (α-MEM: Sigma Chemical Co., St. Louis, MO, USA) containing 10% fetal bovine serum (FBS: Filtron, Brooklyn, Australia), 100 µg/mL ascorbic acid (Wako Pure Chemical Industries, Osaka, Japan), 1× Glutamax^®^ (Invitrogen, Carlsbad, CA, USA), 100 U/mL penicillin (Invitrogen) and 100 µg /mL streptomycin (Invitrogen). The cultures were maintained at 37 °C in a humidified atmosphere of 5% CO_2_ in air and the medium was changed twice a week.

### 4.5. Semi-Quantitative Reverse Transcription-Polymerase Chain Reaction (RT-PCR)

DP-805 cells were seeded at a density of 1 × 10^5^ cells/dish into 60 mm culture dishes (Becton Dickinson Labware, Fraklin Lakes, NJ, USA) and cultured until they reached confluence. In this study, the day when cells reached confluence was designated as day 0. At 3 days before reaching confluence (−3 days), the cells reached ~70% of confluence. The expression of mRNA encoding Mel1aR in DP-805 cells was examined by reverse transcription-polymerase chain reaction (RT-PCR) at −3, 0, 1, 3, 5, 7, 14 and 21 days of culture. Total RNA was extracted using TRIzol^®^ reagent (Invitrogen). cDNA synthesis was performed from 1 µg total RNA using the Superscript III First-Strand Synthesis System (Invitrogen) after DNase I (Invitrogen) treatment. PCR was carried out in a 50 µL reaction mixture using Thermo ReddyMix PCR Master Mix (Thermo Fisher Scientific, Braunschweig, Germany). Conditions and primer sequences for PCR amplification are shown in Table 1. The glyceraldehyde-3-phosphate dehydrogenase (*GAPDH*) gene was used as an internal control for the quantity and quality of cDNA. PCR products were separated in a 2% agarose gel and visualized under UV light after ethidium bromide staining.

**Table 1:** Oligonucleotide primers used in RT-PCR.

Primer (GenBank Accession Number)	Sequences	Size (bp)	Annealing Temperature	Cycles
human Mel1aR	F: 5'-GATCCTGGTTGTCCAGGTCA-3'	241	60	35
(NM_005958)	R: 5'-CATTGAGGCAGCTGTTGAAA-3'			
GAPDH	F: 5'-ACCACAGTCCATGCCATCAC-3'	451	56	23
(NM_001289746)	R: 5'-TCCACCACCCTGTTGCTGTA-3'			

F: forward; R: reverse.

### 4.6. Western Blot Analysis

DP-805 cells were seeded into 60 mm culture dishes (Becton Dickinson Labware) at a density of 2 × 10^3^ cells/dish. After reaching confluence, the cells were lysed with RIPA buffer (10 mM Tris–HCl, 1% NP-40, 0.1% SDS, 150 mM NaCl and 1 mM EDTA) containing protease inhibitor cocktail (Thermo Fisher Scientific, Rockford, IL, USA). The homogenates were centrifuged at 10,000 rpm, 4 °C for 10 min. The supernatants were mixed with the NuPAGE LDS sample buffer (Invitrogen) and heated for 3 min at 100 °C. The samples (100 µg protein) were electrophoretically separated by the NuPAGE System (Invitrogen) using a 4%–12% Bis–Tris gel, and electroblotted onto a polyvinylidene difluoride (PVDF) membrane (Invitrogen) using an iBlot Dry Blotting System (Invitrogen). The membrane was blocked with Western Breeze Blocking Solution (Invitrogen) for 30 min at RT, and incubated with 1 µg/mL of mouse anti-melatonin 1a receptor monoclonal antibody (1:1000; Abnova Corporation) or rabbit anti-β-actin antibody (1:1000; Biolegend, San Diego, CA, USA) at 4 °C overnight. After several rinses with Western Breeze Wash Solution (Invitrogen), the membrane was incubated with Western Breeze Secondary Antibody Solution (Invitrogen) for 30 min. After several washes, Mel1aR and β-actin were visualized using Western Breeze Chemiluminescent Substrate (Invitrogen) and an enhanced chemiluminescence (ECL) mini-Camera (Amersham Pharmacia Biotech, Poole, UK).

### 4.7. Cell Proliferation Assay

The effect of melatonin on the proliferation of DP-805 cells was analyzed using a crystal violet (Sigma) staining method. The DP-805 cells were seeded at a cell density of 1 × 10^4^ cells/well in 24-well culture plates (Becton Dickinson Labware) and cultured in α-MEM containing 10% FBS and various concentrations (0, 1, 10, 50, 100 and 200 µM) of melatonin (Sigma) for 1, 3, 5, 7, 10 and 14 days. In each scheduled day, cells were rinsed with PBS and fixed with 1% glutaraldehyde (Wako) in PBS at 4 °C overnight. Thereafter, the cells were stained with 0.02% crystal violet (Wako) in deionized water for 30 min. After several rinses with distilled water, crystal violet bound to cells was extracted by the incubation with 500 µL/well of 70% ethanol at 4 °C overnight. Absorbance was measured at 570 nm using a microplate reader xMark (BIO-RAD Laboratories Inc., Carlsbad, CA, USA).

### 4.8. Real-Time Reverse Transcription-Polymerase Chain Reaction

The expressions of mRNA encoding OPN, OCN, BSP, DMP-1 and DSPP in DP-805 cells were examined by real-time PCR. PCR was performed with SYBR^®^ Premix ExTaqII™ (Takara Bio Inc., Shiga, Japan) using an Applied Biosystems StepOne™ Real-Time PCR System (Applied Biosystems Inc., Carlsbad, CA, USA). Conditions and primer sequences for PCR amplification are shown in Table 2. The *GAPDH* gene was used as an internal control for the quantity and quality of cDNA.

**Table 2:** Sequences of primers used in real-time RT-PCR.

Primers (GenBank Accession Number)	Sequences	Size (bp)
OPN	F: 5'-TGAAACGAGTCAGCTGGATG-3'	162
(NM_001251830)	R: 5'-TGAAATTCATGGCTGTGGAA-3'	
OCN	F: 5'-GTGCAGAGTCCAGCAAAGGT-3'	175
(NM_001199662)	R: 5'-TCAGCCAACTCGTCACAGTC-3'	
BSP	F: 5'-CAACAGCACAGAGGCAGAAA-3'	247
(NM_004967)	R: 5'-CGTACTCCCCCTCGTATTCA-3'	
DMP-1	F: 5'-CAGGAGCACAGGAAAAGGAG-3'	212
(NM_004407)	R: 5'-CTGGTGGTATCTTGGGCACT-3'	
DSPP	F: 5'-TCACAAGGGAGAAGGGAATG-3'	181
(NM_014208)	R: 5'-TGCCATTTGCTGTGATGTTT-3'	
GAPDH	F: 5'-GAGTCAACGGATTTGGTCGT-3'	261
(NM_001289746)	R: 5'-TTGATTTTGGAGGGATCTCG-3'	

F: forward; R: reverse.

## 4.9. Alizarin Red S Staining

The effect of melatonin on the mineralized matrix formation was examined by Alizarin Red S (Wako) staining. DP-805 cells were seeded at a cell density of 1 × 10^5^ cells/well in 6-well culture plates (Becton Dickinson Labware) and cultured. At confluence, the medium was changed to α-MEM supplemented with 10% FBS, 100 µg/mL ascorbic acid, 1× Glutamax^®^, 100 U/mL penicillin (Invitrogen), 100 µg /mL streptomycin, 10 mM of β-glycerophosphate (Wako), 10^−7^ M dexamethasone (Sigma) and various concentrations (0, 1, 10, 50, 100, 200 µM) of melatonin. At 28 days after confluence, the cells were fixed with 1% glutaraldehyde for 15 min at RT and stained with 10 mg/mL of Alizarin Red S solution.
